# Melatonin as a Reducer of Neuro- and Vasculotoxic Oxidative Stress Induced by Homocysteine

**DOI:** 10.3390/antiox10081178

**Published:** 2021-07-24

**Authors:** Kamil Karolczak, Cezary Watala

**Affiliations:** Department of Haemostatic Disorders, Medical University of Lodz, ul. Mazowiecka 6/8, 92-215 Lodz, Poland; cezary.watala@umed.lodz.pl

**Keywords:** melatonin, homocysteine, oxidative stress, neurotoxicity, cardiovascular diseases

## Abstract

The antioxidant properties of melatonin can be successfully used to reduce the effects of oxidative stress caused by homocysteine. The beneficial actions of melatonin are mainly due to its ability to inhibit the generation of the hydroxyl radical during the oxidation of homocysteine. Melatonin protects endothelial cells, neurons, and glia against the action of oxygen radicals generated by homocysteine and prevents the structural changes in cells that lead to impaired contractility of blood vessels and neuronal degeneration. It can be, therefore, assumed that the results obtained in experiments performed mainly in the in vitro models and occasionally in animal models may clear the way to clinical applications of melatonin in patients with hyperhomocysteinemia, who exhibit a higher risk of developing neurodegenerative diseases (e.g., Parkinson’s disease or Alzheimer’s disease) and cardiovascular diseases of atherothrombotic etiology. However, the results that have been obtained so far are scarce and have seldom been performed on advanced in vivo models. All findings predominately originate from the use of in vitro models and the scarcity of clinical evidence is huge. Thus, this mini-review should be considered as a summary of the outcomes of the initial research in the field concerning the use of melatonin as a possibly efficient attenuator of oxidative stress induced by homocysteine.

## 1. Introduction

Melatonin, a hormone secreted by the pituitary gland, is known primarily for its involvement in the regulation of the circadian biorhythm [1,2]. In recent years, new properties of melatonin are still being discovered, which widens our view on the action of this tryptophane derivative in the human body and shows that the classic melatonin effects of influencing the sleep and wakefulness phases are directly associated with inflammation, balanced energy metabolism, cardioprotection, and more [3,4,5,6]. One of the most currently studied features of melatonin is its capability to reduce oxidative stress [7].

Homocysteine is a thiol amino acid that is an intermediary in the metabolism of cysteine and glutathione [8]. Since the discovery of increased atherogenesis in homocystinuria, homocysteine has had our attention as a possible factor perpetuating atherogenesis [9] and neurotoxicity [10], not only in some rare genetic conditions, but also under a hyperhomocysteinemic state caused by an imbalanced diet [11,12], some drugs [13], or the course of aging [14]. Biotoxic effects of homocysteine are mainly attributed to its oxidative action in cells [15].

**Figure 1:**
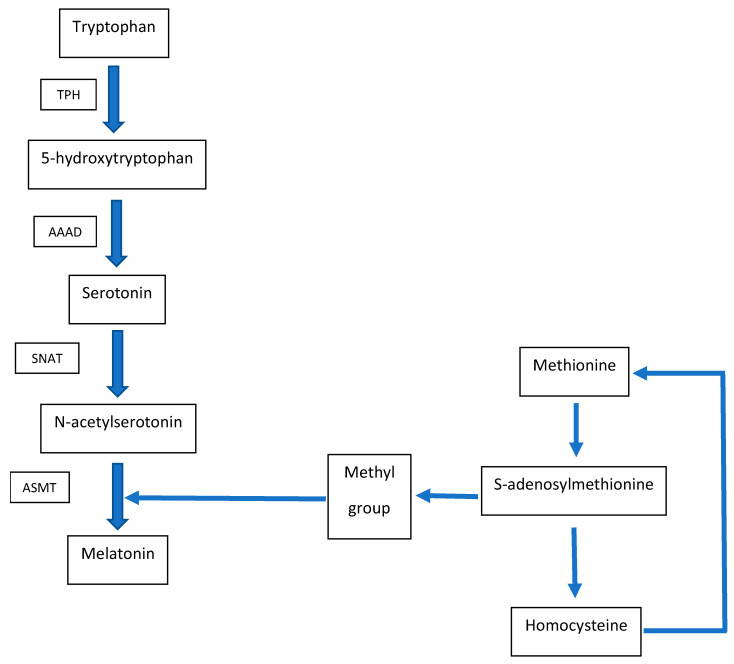
Biochemical interconnection between homocysteine and melatonin metabolism. TPH, tryptophan hydroxylase; AAAD, aromatic amino acid decarboxylase; SNAT, serotonin *N*-acetyltransferase; ASMT, *N*-acetylserotonin *O*-methyltransferase.

Interestingly, the metabolisms of melatonin and homocysteine are interconnected (Figure 1). One of the stages of melatonin synthesis is the methylation of acetylserotonin. The methyl group donor in this reaction is S-adenosylmethionine, and the by-product is S-adenosylhomocysteine, which is further converted to homocysteine [16]. Homocysteine is a low-molecular-weight thiol that has a non-specific effect on cells. It is a molecule with a wide range of activities in the human body [15], and the number of disorders leading to various diseases associated with the state of hyperhomocysteinemia is quite high [17,18]. From a social point of view [19,20], the two most important groups of diseases associated with disorders of homocysteine metabolism are diseases of the nervous system, especially those of neurodegenerative etiology [10], and cardiovascular diseases of thrombotic etiology [21]. These diseases usually coexist [22], especially in the elderly, and also show a common biochemical basis, which is the non-specific induction of oxidative stress by homocysteine [10,23]. The reactive species of oxygen generated in homocysteinylated cells trigger further processes that can lead to neuronal or endothelial apoptosis [24,25], the activation of platelets [26], the stimulation of myocyte proliferation [27], the oxidation of LDL particles [28], and so forth. The initiators of these reactions are always reactive oxygen species, which react with the lipids and proteins of cells, leading to an excessive accumulation of oxidized biomolecules and structural alterations of cells [17]. Thus, the effective lowering of homocysteine levels appears to be a promising mechanism to prevent redox imbalance and to decrease the occurence of neurodegenerative and thromboembolic diseases, often coexisting, especially in the elderly. Since the metabolism of low -molecular-weight thiols depends on the efficient operation of a number of enzymes, the maintenance of the appropriate concentration of the cofactors (folic acid, vitamins B6 and B12) of these enzymes should be a promising way to keep the state of normohomcysteinemia [17,18,29,30]. Unfortunately, in some studies, food fortification with the mentioned cofactors has not been shown to effectively restore physiological levels of homocysteine [31,32]. Moreover, the consumption of food rich in these ingredients is highly diversified depending on the local diet in a given geographical region, along with age, sex, and other factors, and is sometimes insufficient [33], especially among the already mentioned elderly population [34] who are particularly exposed to homocysteine-dependent oxidative stress and the development of thromboembolic and neurodegenerative diseases. Therefore, there is a need to look for new agents that can counteract the toxic effects of homocysteine. Good candidates seem to be antioxidants, which, as nonspecific molecules, can inhibit homocysteine-dependent oxidative stress already in the initial phase of the pathological path, that is, at the stage of generation of oxygen free radicals [35,36,37,38].

The choice of potential antioxidant molecules is very wide. One of the best known is endogenous melatonin, which has shown the ability to scavenge oxygen free radicals [39] and to stimulate an enzymatic antioxidant defense [40]. Its pharmacological profile is known and the safety of its use does not raise any serious concerns among researchers and clinicians [41]. Perhaps melatonin, a molecule with a well-known (although much remains to be investigated) antioxidant activity, will prove to be an effective limiter of the oxidative stress induced by homocysteine and, thus, a significant reducer of the risk of the development of homocysteine-dependent neurodegenerative and thromboembolic diseases co-occurring, as often mentioned, especially in elderly people. Given the increasing life expectancy and the increasing proportion of elderly people in the world population, the melatonin route of reducing the toxic effects of homocysteine may become a promising way to improve the quality of life, reduce the risk of hospitalization, disability, and dependence on others in the elderly, as well as reducing the burden on health systems through costly life-saving procedures.

Thus, let us take a look at what the published data can tell us about melatonin’s potential ability to inhibit the toxic effects of homocysteine, especially in the brain and vascular system.

## 2. Metabolic Interconnection between Melatonin and Homocysteine

A folate deficiency in the diet leads to a state of hyperhomocysteinemia [14,42]. However, so far, no one has asked how the folate-dependent hyperhomocysteinemia will affect melatonin levels, and even more so, considered the fact that the pathways of metabolism of low-molecular-weight thiols and melatonin synthesis have been shown to be tangled together. When such a question has been asked, it turned out that hyperhomocysteinemia caused by a lack of folates is accompanied by decreased melatonin levels in the pituitary gland, and, thus, also decreased the concentrations of melatonin metabolites excreted in urine [16]. This is clear evidence showing that the link between low-molecular-weight thiols and melatonin synthesis pathways through a methyl group is biochemically reflected in both the brain and peripheral tissues. Therefore, it should be expected that a folate deficiency in the diet, which is quite common in some subpopulations [43], can seriously affect the efficiency of melatonin synthesis in the pituitary gland. Given the multiplicity of the physiological effects exerted by melatonin, the consequences of such a deficit in the production of the protective factor (melatonin), with the concomitant increase in the concentration of the pathogenic factor (homocysteine), both “working” in a tissue-non-specific manner, can be quite serious for the organism’s homeostasis.

In the following sections of this article, we discuss the possibility of the prevention against homocysteine-induced cell changes by melatonin based on the experimental models, in which melatonin added as an exogenous agent mainly played the role of a scavenger of oxygen free radicals generated by homocysteine. It is worth emphasizing at this point that we have a reason to believe that melatonin is not only a scavenger of oxygen radicals induced by homocysteine, but also acts as a hormonal factor that regulates the metabolism of low-molecular-weight thiols, including homocysteine. This can be judged by looking at the experimental animals subjected to pinealectomy, in which, after removing the cellular source of melatonin, the levels of homocysteine in the blood considerably increased [44]. The mechanism of this phenomenon is completely unclear, but it is in line with other reports indicating that homocysteine levels are under hormonal regulation by the hormones of various endocrine glands [45,46,47,48]. The changes in homocysteine concentrations due to pituitary removal can be counteracted by the administration of exogenous melatonin. In addition, the view that modulation of homocysteine metabolism by melatonin as the superior hormonal factor is supported by the observations showing that the daily fluctuations in the blood levels of homocysteine show a rhythm consistent with the daily rhythm of the fluctuations in the blood levels of melatonin, that is, with a peak at 2 a.m. and a nadir at 2 p.m. Interestingly, this characteristic rhythm persists even when the pituitary gland is surgically removed, and thus, the homocysteine levels reduced by a pinealectomy maintain the described diurnal variability [44]. Hence, can we conclude that this is a melatonin-dependent process since it does not disappear after pituitary removal? Unfortunately, we have no unambiguous evidence at present to answer this question.

Similarly, the nocturnal peak for homocysteinemia, along with a mid-day or morning nadir, was observed by other research teams, confirming the above-mentioned results [49,50]. Another team of researchers detected an acrophase of melatonin concentration in juvenile rats at 1 p.m., noting that the diurnal fluctuations in homocysteinemia disappeared in older rats. Surprisingly, however, the oldest rats demonstrated lower levels of homocysteine than the middle-aged animals [51], which would contradict the generally accepted model of homocysteinemia increasing with age [14]. Thus, the issue of the circadian rhythm of homocysteinemia and its shaping by melatonin seems to be a controversial issue based on the gathered data and certainly requires further, more extensive experimental verification.

## 3. Melatonin and Homocysteine in Neurotoxicity

The brain is a structure that is particularly susceptible to oxidative damage. This susceptibility shows some regional variation, and different subpopulations of neurons “enclosed” in one anatomical structure, for example, in the hippocampus, cerebellum, or the black substance, may be characterized by different abilities to cope with reactive oxygen species [52]. In general, the susceptibility of brain cells to oxidative stress is caused by several biochemical features, such as the accumulation of unsaturated fatty acids, high concentrations of transition metal ions, the production of superoxide anion by microglia, a relatively poor antioxidant defense, high metabolic cell activities (especially of mitochondria) and the high level of the metabolism of neurotransmitters [53]. The exposure of such sensitive brain cells to homocysteine can disrupt the delicate redox balance, thus contributing to disturbances at the cellular level that may lead to a clinical manifestation. We know that the substantia nigra [54,55], the hippocampus [56,57,58], the cerebral cortex [58,59], as well as the cerebellum, that is, key structures for the pathogenesis of Parkinson’s disease [60], Alzheimer’s disease [56,58], or fetal alcohol syndrome [61], are preferable sites of the neurotoxic action of homocysteine in the brain [62,63,64]. Thus, researchers look for efficient anti-homocysteinemic molecules that plausibly reduce the homocysteine-induced oxidative damage in the brain. One of the molecules attracting some attention as a potential inhibitor of the homocysteine-induced oxidative damage in these structures is melatonin.

Simple in vitro models have shown that proteins and lipids in rat brain homogenates are effectively protected by melatonin against homocysteine-induced oxidation [65,66]. These experiments were further developed into interesting in vivo animal models related more directly to human neurodegenerative disorders.

Homocysteine administered directly to the substantia nigra caused an increase in the concentration of reactive oxygen species (especially the hydroxyl radical), a decrease in the level of glutathione, and an increase in the activity of antioxidant enzymes. Among the cellular substructures, homocysteine showed particular toxicity to mitochondria. Interestingly, melatonin administered peripherally (intraperitoneally) successfully reversed the harmful effects of the intracerebrally-injected homocysteine. Importantly, the salvage effect of melatonin was visible not only at the molecular level as a decrease in the concentration of free oxygen radicals, as well as an increase in glutathione concentration, normalizations of antioxidant enzyme activities, and prevention of dopamine degradation, but also at the functional level, including an improvement in a rotational behavioral bias [67]. The results of this study can be considered important because they prove that biochemical changes in the substantia nigra caused by homocysteine can be effectively inhibited by peripherally administered melatonin. From the pharmacological point of view, this is a very important notion. So far, however, the use of melatonin as a potential drug in Parkinson’s disease has been studied mainly in animal models, where numerous beneficial effects of melatonin have indeed been observed [68,69], but with no reference to eventual changes in homocysteinemia. Homocysteine is simply not considered a potential mediator in the pathogenesis of these models, so the beneficial effects of melatonin are not associated by the authors with the anti-homocysteine effect. In human studies, in turn, involving patients suffering from Parkinson’s disease, the effects of melatonin are sometimes questionable for purely methodological reasons, regarding the way the experiment was conducted [70]. Most studies involving humans subjects with Parkinson’s disease do not go beyond examining the classic effects of melatonin, and hence, they are limited to assessing the changes in the phases of sleep and wakefulness in this group of patients [71,72,73], again, with no reference to homocysteine and its neurotoxic potential. Relatively new results, presented by Paul et al. [70], may, in the nearest future, open the way to animal and human trials using melatonin as a therapy to support the treatment of Parkinson’s disease, with particular emphasis on the anti-homocysteine effect. These studies, to provide unambiguous outcomes, must be well-designed because, among the overwhelming multitude of reports on the therapeutic effect of melatonin, there are also those claiming that melatonin may worsen the course of experimental models of Parkinson’s disease [74,75]. In future research that focuses on the antagonization of hyperhomocysteinemia by melatonin, it is worth also taking this unfavorable possibility into account.

Hyperhomocysteinemia can be induced locally by the administration of a homocysteine solution directly to the selected brain structure [70]. In addition, general hyperhomocysteinemia can be induced by the dysregulation of homocysteine metabolism, for example, by the administration of methionine in pure drinking water to laboratory animals [76,77]. In this model, we also deal with the neurotoxicity of homocysteine and the neuroprotection exerted by melatonin. Such methionine-induced hyperhomocysteinemia leads to oxidative stress (lipid peroxidation and decreased glutathione peroxidase activity) and the hyperactivity of glial cells in the hippocampus and cortex [77].

Another important factor causing hyperhomocysteinemia is ethyl alcohol taken chronically [78], or even moderately on some social occasions [79]. However, not all types of alcoholic beverages can equally affect homocysteine levels. For example, whiskey is homocysteinogenic, while wine does not affect homocysteine levels [80]. Alcohol administered to females during pregnancy can induce hyperhomocysteinemia in their offspring. Again, the organ particularly exposed to oxidative stress in the fetuses will be the developing brain, especially the cerebellum, and the observed molecular and functional changes may serve as a model of the fetal alcohol syndrome. In the offspring of female rats that were treated with ethanol and melatonin during pregnancy, the development of hyperhomocysteinemia in the cerebellum was inhibited and the lipid peroxidation process was reduced, which, in turn, resulted in less severe motor defects in the newborn rats, in comparison to the offspring born of the females treated with ethanol, but without the melatonin supplementation [81]. This single report presents significant results pointing to homocysteine as an important element in the pathogenesis of motor disorders in fetal alcohol syndrome, and to melatonin as an effective preventive factor.

Melatonin prevents not only pathological changes in motor functions caused by homocysteine, but also protects cognitive functions and memory. The melatonin-induced reduction in the level of oxidative stress (expressed in an increased level of lipid peroxidation and a reduced level of glutathione), previously induced by the action of homocysteine, allows for keeping two pivotal cognitive functions: learning and memory performance, mainly due to the maintenance of synaptic plasticity, especially in the area of the hippocampus [82].

While discussing the evidence gathered so far and showing the possibilities of the use of melatonin in reducing the pathology of homocysteine-dependent brain diseases, it is also necessary to mention psychiatric diseases. We have data proving that the level of homocysteine is elevated in such diseases as depression [83], schizophrenia [84], or bipolar disorder [85]. The importance of homocysteine in the development of these diseases is still debated. There are no unambiguous reports in the literature on the effects of melatonin on the homocysteine levels in psychiatric diseases. We only know that in the group of patients with the first episode of depression, both the increased levels of homocysteine and the elevated levels of melatonin are observed concomitantly, but it is not yet known to which extent these increases are causally related [80]. We can hypothesize that the simultaneous increase in the concentrations of an antioxidant and an oxidant [86] might be a protective mechanism against the action of the latter one. However, in some conditions, such as ulcerative colitis [87] or a herpes zoster infection [88], the state of hyperhomocysteinemia is accompanied by a decreased melatonin concentration. Thus, in these cases, there would be no compensatory antioxidant response (melatonin) to the oxidant (homocysteine), as we might have speculated to occur in depression. Perhaps, however, it is the result of some specific conditions related to these diseases. Further research is needed to shed more light in this field.

With just a few available studies, it is not possible to solve the question of what the real cause of the antihomocysteine effect of melatonin in brain diseases is, or whether such a putative effect would be relevant to the findings originating from clinical observations. Certainly, the role of melatonin as a scavenger of free radicals generated by homocysteine in the nervous tissue seems to be the most probable mechanism. The influence on the expression of glutathione peroxidase cannot be ruled out due to the repeated observations of the increased glutathione concentration in cells exposed to melatonin under the conditions of hyperhomocysteinemia (both in the brain as well as in peripheral tissues). This effect may be not only due to the fact that melatonin undertakes the functions of the anti-radical shield “saving” the glutathione from oxidation, but it is also due to the increased glutathione peroxidase expression. We know that such regulation by melatonin at the level of the transcription of the gene encoding glutathione peroxidase takes place in the brain [40].

**Figure 2:**
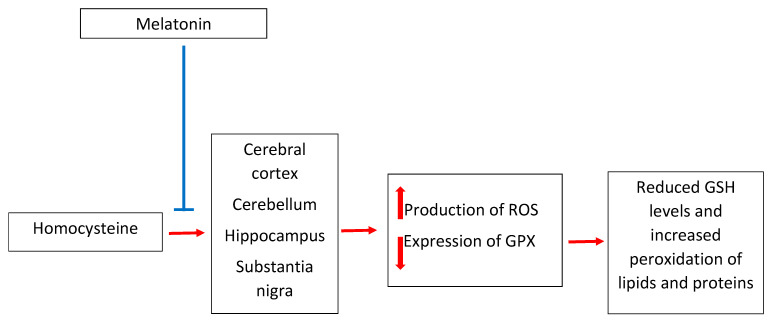
Melatonin inhibits the neurotoxicity of homocysteine. In regions of the brain particularly sensitive to oxidative stress, homocysteine increases the synthesis of reactive oxygen species, which react more intensively with glutathione, leading to a reduction in the level of this endogenous antioxidant. In addition, a reduction of glutathione peroxidase expression does not allow for an effective reduction of lipid hydroperoxides and hydrogen peroxide. This results in oxidative damage to brain lipids and proteins, the accumulation of which can lead to clinical symptoms of Parkinson’s disease, Alzheimer’s disease, and fetal alcohol syndrome. Melatonin prevents these adverse homocysteine-induced changes by scavenging the reactive oxygen species induced by homocysteine and by direct positive regulation of glutathione peroxidase expression. GSH, glutathione; GPX, glutathione peroxidase; ROS, reactive oxygen species.

Thus, the neuroprotective effect of melatonin (Figure 2) in hyperhomocysteinemia-related brain disorders has at least several complementary points of action, which may determine the presumed neuropharmacological efficacy of the pineal hormone. However, this conclusion is drawn on the basis of a single report. It is quite surprising that a huge amount of studies investigating the effects of melatonin on various systems and a large number of studies on homocysteine share such a small area of overlap and that the field of the neuroprotection triggered by melatonin under the state of hyperhomocysteinemia is so poorly explored. It should be added, herein, that in the work done to this date, the range of research methods used to assess the markers of oxidative stress is quite repeatable and rather not advanced. Still, the detection of malondialdehyde as a marker of lipid peroxidation is very popular, which, due to numerous technical drawbacks, generates rather low-reliability outcomes [89]. Therefore, very generally speaking, we are at the early stage of research on the possible use of melatonin as an agent reducing the neurotoxicity of homocysteine.

## 4. Melatonin and Homocysteine in Cardiovascular Diseases: Focus on Endothelium

Homocysteine is considered to be an important contributor to the increased risk of the development of atherosclerosis [90]. In this respect, the endothelium is believed to be one of the major targets of homocysteine [91]. Additionally, in this case, it is commonly accepted that the reactive oxygen species generated by homocysteine are of key importance for the acceleration of atherogenesis [92].

**Figure 3:**
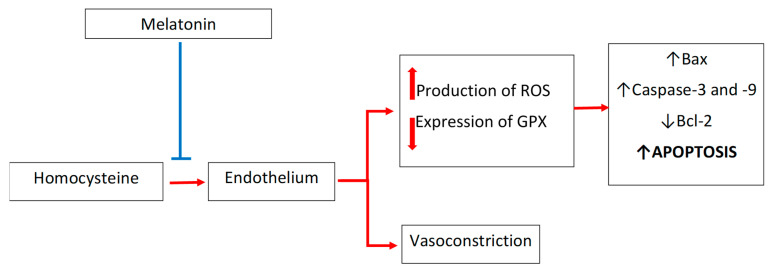
Melatonin prevents the vasculotoxicity of homocysteine. In endothelial cells, homocysteine enhances the synthesis of reactive oxygen species, which react intensively with glutathione, leading to a reduction in the level of this endogenous antioxidant. Simultaneously, a reduction of glutathione peroxidase expression does not allow for an effective reduction of lipid hydroperoxides and hydrogen peroxide. As a result of increased oxidative stress, the motility of the blood vessel wall is disturbed, vasoconstriction is increased, and the ability to relax is impaired. Reactive oxygen species initiate endothelial cell apoptosis. Melatonin prevents homocysteine-dependent pathological changes by directly scavenging reactive oxygen species and by stimulating glutathione peroxidase expression. Bax, bcl-2-like protein 4; Bcl-2, B-cell lymphoma 2; GPX, glutathione peroxidase; ROS, reactive oxygen species.

The intense oxidative stress observed in homocysteinylated endothelial cells leads to cell apoptosis that is dependent on the changes in the cellular expressions of caspase-3, caspase-9, Bax, and Bcl-2. Melatonin effectively reverses these changes and shows anti-apoptotic effects; it lowers the expressions of caspase-3, caspase-9, and Bax protein, and increases the expression of the Bcl-2 protein, which is the direct consequence of the melatonin-mediated scavenging of reactive oxygen species in the homocysteinylated endothelium. Endothelial cells incubated with homocysteine and melatonin showed much greater viability compared to cells incubated with homocysteine alone, without melatonin [93,94]. Interestingly, in an experimental model using KCl as a vasoconstrictive factor, homocysteine has been shown to exaggerate the effect of KCl; however, homocysteine-augmented vasoconstriction was reduced by melatonin [95] [Figure 3].

Since blood vessel walls are histologically complex structures, the effects exerted on the functioning of the endothelium are also reflected in the physiology of other and deeper layers of the vascular wall. The hydroxyl radicals generated by homocysteine reduces the pool of bioavailable nitric oxide. The decreased concentration of this important vasodilating factor increases the contractility of smooth muscles [95,96,97]. In both cases, homocysteine has a disruptive effect on the vasomotor properties of blood vessels, contributing to further atherogenic changes. Melatonin, although unable to prevent the oxidation of homocysteine itself, especially in the presence of Fe^2+^ ions, has effectively reversed the effects of homocysteine and reduced the exaggerated contractility of homocysteinylated vessels [95,96,97].

These results strongly suggest that the atherogenic effect of homocysteine on endothelium- and smooth muscle cell-dependent vessel contractility and dilatation may be counteracted by melatonin. This topic, which has only been very superficially touched upon by the mentioned papers, should certainly be explored in further studies.

## 5. Melatonin as a Regulator of Blood Homocysteine Levels

Particularly intriguing is the question of whether melatonin may be active even before homocysteine would trigger oxidative stress. We should assume that for such an early action, melatonin would have to regulate homocysteine synthesis and, thus, influence its level in the blood. Do we have reasons to consider such a hypothesis likely? Indeed, we have. In the methionine model of hyperhomocysteinemia, in which animals were watered with solutions of this thiol amino acid instead of pure drinking water, the simultaneous administration of melatonin inhibited the increase in homocysteine concentration, which, in turn, reduced the level of lipid peroxidation in the brain [76]. Another research group, using the same experimental model (hyperhomocysteinemia induced by the administration of methionine in drinking water), observed significantly decreased homocysteine concentrations in the circulating blood as caused by the simultaneous melatonin intake. Although melatonin did not normalize the homocysteine concentrations to control levels (melatonin was not so efficient in such an amelioration, as was the withdrawal of methionine itself) and the concentration of homocysteine was still within the pathological ranges, it was, on average, two-fold lower than in the animals treated with methionine alone. This effect appeared after a longer (two months), but not a shorter (one month) subcutaneous administration of melatonin. Interestingly, the two-month hyperhomocysteinemia was not accompanied by increased concentrations of lipid peroxidation products in blood plasma, nor was it characterized by decreased nitric oxide concentrations [98]. Unfortunately, as it seems, the imprecise planning of this interesting experiment and the obviously poorly performed statistical analysis (multiple U Mann–Whitney tests instead of analysis of variance) do not allow the results revealed by Murawska-Cialowicz et al. [98] to be regarded as fully reliable.

The action of melatonin as a regulator of homocysteine levels in the body can be specifically attributed to this pineal neurohormone. Other tested antioxidants, such as vitamin C, lipoic acid, or vitamin E, have not demonstrated such a property [99]. Importantly, the lowering of the homocysteine levels by melatonin was also observed in animals fed a diet rich in fructose, used as an alternate model of hyperhomocysteinemia [100]. These findings may be of particular importance in the context of the attempts to lower the concentrations of homocysteine in patients with hyperhomocysteinemia and some coexisting disorders of glucose and lipid metabolism.

Overall, it seems that melatonin can act not only as a scavenger of the homocysteine-induced free radicals under conditions of hyperhomocysteinemia, but it can also normalize the homocysteine concentration itself. This conclusion is based, however, merely on singular reports in which only the model of the methionine-induced hyperhomocysteinemia has been tested. Therefore, there is an indisputable need to further verify the effect of melatonin on blood homocysteine levels in both the methionine model and other dietary or genetic models of hyperhomocysteinemia.

It should be emphasized that the current state of knowledge does not allow us to even briefly describe the exact molecular mechanisms responsible for the ability of melatonin to counteract homocysteine toxicity dependent on reactive oxygen species, either in the central nervous system or the endothelium, as well as in other tissues. The data collected above provides only superficial proof that melatonin actually reduces the oxidative stress induced by homocysteine, but does not allow it to bind to specific cellular proteins and biochemical pathways.

One of the first proteins that comes to mind when we think of cellular oxidative stress is NADPH oxidase (NOX)—an enzyme capable of generating superoxide anions and other reactive oxygen derivatives. These radicals are involved in both physiological reactions (such as phagocytosis, gene expression regulation, and post-translational modifications of proteins) and pathological processes, including those related to neurodegeneration and thrombosis [101]. It seems, however, that this protein should not be considered the intracellular point at which the biochemical battle between melatonin and homocysteine takes place. Admittedly, there is evidence that both melatonin [102,103,104] and homocysteine [105,106,107] reduce and increase, respectively, the generation of reactive oxygen species by NOX. However, there are no reports that investigate whether the inhibition of the pro-oxidative activity of NOX in the cell by melatonin contributes significantly to the defense of cells against the toxicity of homocysteine, known as a stimulator of the activity of this protein. The hypothesis that inhibition of the pro-oxidative activity of NOX by melatonin may counteract homocysteine toxicity is interesting, but it is also somewhat controversial because other inhibitors of NOX activity do not counteract homocysteine-induced disorders, and even aggravate them [108,109]. Presently, it is unknown what eventual role NOX protein (and its subunits) plays in the protection of cells by melatonin against homocysteinylation.

## 6. Conclusions

The presented literature review allows us to answer the question posed in the title of the article affirmatively: indeed, melatonin prevents pathological changes induced by homocysteine. This positive response should, however, be subject to certain reservations that will cause it to be treated with necessary caution and, more and more, as an encouraging prospect for further experimental exploration of the granted affirmative. The available literature on the discussed topic is not abundant, and numerous papers have been published repeatedly by the same research teams. It is clear that this is not a popular subject of investigation, even though the research on melatonin on one side and on homocysteine on the other (investigated separately and disconnectedly) is generally very abundant. What has been summarized in this paper strongly encourages the exploration of the use of melatonin in the prevention of the effects of hyperhomocysteinemia more intensively, as it may have important clinical effects in the prevention of thromboembolic and neurodegenerative diseases.

In future research, more experiments should be undertaken in animal models of hyperhomocysteinemia and thromboembolic and neurodegenerative diseases, which are available in numerous and diverse variants. It is also worth undertaking research involving humans even more so, considering the fact that melatonin has already been widely used in medical practice for decades.

Clinical trials in the discussed field would be an extremely valuable and even necessary supplement to the rather modest knowledge accumulated so far from in vitro and animal models.

It also seems necessary to expand the experimental methods, significantly widening the knowledge of the molecular aspects of the interaction of homocysteine and melatonin, especially taking into account that this knowledge is currently only rudimentary. In addition, we should remain aware that the topic does not only come down to the protective effect of melatonin in homocysteinylated tissues on alterations to the concentrations of reactive oxygen species and malondialdehyde.
